# Hyperuricemia Is Not an Independent Predictor of Erectile Dysfunction

**DOI:** 10.1016/j.esxm.2020.100319

**Published:** 2021-02-20

**Authors:** Antti T. Tuokko, Teemu Murtola, Päivi Korhonen, Antti Kaipia

**Affiliations:** 1 Department of Urology, Tampere University Hospital, Tampere, Finland; 2 Tampere University, Faculty of Medicine and Health Technology, Tampere, Finland; 3 Department of Surgery, Seinäjoki Central Hospital, Seinäjoki, Finland; 4 Department of General Practice, Turku University and Turku University Hospital, Turku, Finland; 5 Central Satakunta Health Federation of Municipalities, Harjavalta, Finland

**Keywords:** Serum Uric Acid, Erectile Dysfunction, Risk Factors, Predictive Marker, Cross-Sectional Study

## Abstract

**Introduction:**

Erectile dysfunction (ED) is strongly associated with physiological and metabolic disturbances, and hyperuricemia has been proposed to predict the onset of ED.

**Aim:**

To investigate if hyperuricemia is an independent predictor for ED when all relevant confounding factors are taken into account.

**Methods:**

This is a cross-sectional study of men aged between 45 and 70 years. The population was well characterized for established cardiovascular risk factors, metabolic syndrome, as well as kidney function, depression, and socioeconomic factors. Analysis was limited to 254 men with complete data and also serum uric acid (SUA) measurements were available. This included 150 men with and 104 without ED. The presence and severity of ED was evaluated using International Index of Erectile Function-5 questionnaire. Risk of ED by SUA level was calculated using univariate and multivariable-adjusted logistic regression. Effect modification by participant characteristics were evaluated in subgroup analyses.

**Main Outcome measures:**

The main outcome measures of this study are prevalence and severity of erectile dysfunction.

**Results:**

Patients with ED (59% of the study population) were older than men without ED (59 vs 54 years) and had lower serum testosterone (14.3, 95% CI 11.3–17.3 vs 15.1 nmol/l, 95% CI 12.1–18.8, respectively). Regarding all other variables, the groups were comparable. No significant difference was found for SUA by ED. SUA was not associated with ED risk in univariate or multivariable analysis (multivariable-adjusted OR 1.14, 95% CI 0.59–2.19, *P* = .7) for SUA level higher than median compared with median or lesser (OR 1.00, 95% CI 0.997–1.006, *P* = .7 for continuous variable). No subgroup analysis modified the association. After multivariable adjustment age, education level and depression were statistically significant predictors of ED.

**Conclusions:**

Elevated SUA was not found to be an independent risk factor for ED. Metabolic syndrome, glomerular filtration rate, or cardiovascular risk factors did not modify this result. ED cannot be predicted based on the level of SUA.

**A Tuokko, T Murtola, P Korhonen, et al. Hyperuricemia Is Not an Independent Predictor of Erectile Dysfunction. Sex Med 2021;9:100319.**

## Introduction

Erectile dysfunction (ED) is a major health concern in the aging male population. The prevalence of ED is 10–20% in overall male population and reaches 50–60% at age of 70 years.^1^^,^^2^ In addition to causing distress in itself, it has been associated with onset of cardiovascular disease (CVD) and metabolic syndrome (MetS).^3^^,^^4^ The association of ED with CVD can be largely explained by common risk factors such as aging, diabetes, hypertension, obesity, dyslipidemia, and smoking, but ED is an independent predictor of CVD as well.^5^^,^^6^ The pathophysiology and risk factors of ED and CVD are not yet completely understood.

**Figure 1:**
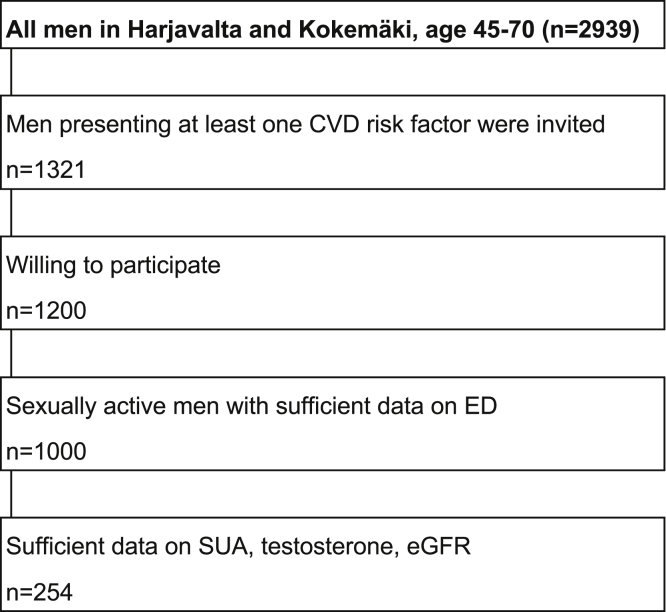
Formation of study population. Cross-sectional analysis of 254 Finnish men with cardiovascular risk factors.

MetS is characterized by central obesity, glucose intolerance, dyslipidemia, and elevated blood pressure, and it shares these risk factors with ED. MetS is also associated with chronic systemic inflammation.^7^, ^8^, ^9^ While the exact pathophysiology underlying systemic inflammation is not known, oxidative stress may play a role. Hyperuricemia, which typically causes oxidative stress, is commonly seen in patients with MetS.^10^^,^^11^ Hyperuricemia can promote small-vessel disease and subsequent disturbance in microcirculation by interfering with nitric oxide signaling and causing smooth muscle proliferation which are commonly seen in ED.^12^^,^^13^ Hyperuricemia is a modifiable risk factor and can be managed through medication and dietary interventions. This could provide new ways to intervene in the development of ED.

In addition to somatic pathophysiology, ED is also influenced by factors affecting libido. Consequently, depression is an established and significant risk factor for ED.^14^^,^^15^ Depression has also been associated with a notable change in serum uric acid (SUA) level.^16^^,^^17^ A clear knowledge gap regarding the association of SUA and ED exists. The effect of glomerular filtration rate (GFR) has been suspected to act as a confounding factor in the association between SUA and ED,^18^ but few studies have been performed on the topic.

We hypothesized that elevated SUA level is independently associated with risk of ED. We used a particularly well-documented group of men of apparent cardiovascular health, where all major cardiovascular risk factors were taken into consideration, including depressive symptoms and estimated GFR (eGFR).

## Methods

This is a cross-sectional study based on the Harmonica Project (Harjavalta Risk Monitoring for Cardiovascular Disease), which was carried out in 2005 to 2007. The study was assessed and approved by the Ethics Committee of Satakunta Hospital Districtm, and all study participants signed their informed consent.

The population consists of all men (n = 2,939) aged between 45 and 70 years and living in the semirural towns of Harjavalta and Kokemäki in Southwest Finland. Men were identified from the national population register center covering the whole of Finland. A cardiovascular risk factor survey and the Finnish Diabetes Risk Score questionnaire were mailed to all home-dwelling men.^19^ Those with prevalent CVD, diabetes, or chronic kidney disease were excluded.

Respondents with a latest measured blood pressure of at least 140/90 mmHg, family history of premature CVD, at least 12 Finnish Diabetes Risk Score in Harjavalta (at least 15 score in Kokemäki) or those using antihypertensive medication were invited for laboratory tests and further examinations. In total, 1,200 men were examined (waist circumference, height, weight, body mass index [BMI], blood pressure) by trained study nurses.

Depressive symptoms were assessed with Beck's depression inventory.^20^ Additional questionnaires regarding alcohol consumption, exercise, and smoking habits were completed. Glucose intolerance was evaluated using a 2-hour glucose tolerance test. eGFR was calculated using the Chronic Kidney Disease Epidemiology Collaboration formula.^21^ Presence of MetS was determined by the The International Diabetes Federation criteria.^22^

The International Index of Erectile Function (IIEF)-5 questionnaire was used to collect data on ED and its severity.^23^ Further 200 men were excluded for no sexual activity (n = 93) or improperly filled IIEF-5 form (n = 107). This included reporting a zero score or leaving any question unanswered. The IIEF-5 questionnaire determines the severity of ED: no ED, 22–25; mild, 17–21; mild to moderate, 12–16; moderate, 8–11; and severe, 5–7. The formation of study population is presented in Figure 1.

The study population, clinical measurements, and laboratory analyses have been previously described in detail by Korhonen et al.^24^ The same cohort data have been used in earlier studies on ED risk factors published by Ettala et al.^25^ High-intensity physical activity, stable relationship status, and schooling after grade school were previously found to be associated with a lower risk of ED. Presence of depressive symptoms increases the adjusted risk of ED in these data.^26^

The present analysis was limited to men with sufficient data on ED severity, based on a correctly answered IIEF-5 questionnaire as well as valid information on age, BMI, use of beta blockers, possible impaired glucose tolerance, and serum levels of total cholesterol, high-density lipoprotein cholesterol (HDL-C), triglycerides, eGFR, SUA, and testosterone. This resulted in a total of 254 cases with complete information available.

### Statistical Analysis

Distribution of risk factors among men with and without ED was compared using Mann-Whitney U-test for continuous variables and Chi-square test for categorical variables.

We first performed age-adjusted analysis for SUA (both continuous variable and stratified by median), BMI, HDL-C, triglyceride, total cholesterol, eGFR (continuous and stratified by median), beta blocker usage, impaired glucose tolerance, systolic blood pressure, smoking, education level, living conditions (living alone/with someone), and depression score to determine possible ED risk factors in our study population.

All variables that were statistically significantly associated with hyperuricemia in univariate analysis were decided a priori to be included in multivariate analysis. In addition, variables related to known clinical risk factors for ED were decided a priori to be included.

Odds ratios and CIs of 95% for the risk of ED were calculated using logistic regression. Age, BMI, eGFR, SUA, testosterone, HDL-C, and triglycerides were first analyzed as continuous variables. Beta blocker medication (any use vs no use), level of glucose tolerance, as well as blood pressure, depression, and education data were entered as categorized variables. To further estimate the role of SUA, testosterone, eGFR, and systolic blood pressure as ED risk factors, we performed analysis where these variables were stratified by median. Continuous variables were used to evaluate risk trends. Odds ratios for ED were estimated using categorical variables.

Subgroup analyses were stratified by potential confounding factors age, BMI, eGFR, testosterone level, impaired glucose tolerance, depression, and presence of MetS.

We calculated a propensity score using logistic regression method with ED as the dependent variable. Variables included in the propensity score were age, BMI, presence of glucose intolerance, blood pressure level, HDL-C, triglycerides and testosterone levels, eGFR, beta blocker usage, and education level. Propensity score was calculated based on odds ratios for ED each of these variables had in the univariate model.

Data were analyzed with SPSS for Mac 24 (SPSS, Inc, Chicago, IL, USA). All *P* values are two-sided.

## Results

### Population Characteristics

ED data were available for a total of 648 men. Majority of them (n = 366, 56.5%) had an ED ranging from mild to severe and 282 (43.5%) men reported no ED. Valid data on SUA were available for 254 patients, and in this group, prevalence of ED was similar at 59%.

Characteristics of the study population are presented in detail in Table 1, categorized by presence of ED. Patients with ED were on average slightly older (59 vs 54 years, *P* for difference <0.001) and had generally lower education level than those without ED (*P* = .002). Glucose intolerance was more common in the ED group but the difference to the non-ED group was not statistically significant (*P* = .538). However, men with ED scored significantly higher in diabetes risk score evaluation (*P* < .001).

**Table 1:** Population characteristics stratified by erectile dysfunction in the population of 254 Finnish men

	ED (n = 150, 59%)	No ED (n = 104, 41%)	*P* For difference
Age (years)∗	59 ± 6	54 ± 6	<.001
Body mass index (kg/m^2^)∗	28.4 ± 3.8	28.8 ± 4.7	.9
Systolic blood pressure (mmHg)∗	149 ± 17.5	147 ± 17.9	.4
Diastolic blood pressure (mmHg)∗	90 ± 8.5	90 ± 9.3	.2
Glomerular filtration rate, estimated (ml/min)†	90 (81–96)	92 (82–101)	.046
Serum uric acid (μmol/l)†	379.4 (336–417)	372.45 (336–402)	.5
Serum uric acid level above median	78 (52%)	49 (47%)	.4
Total cholesterol (mmol/l)†	5.2 (4.6–6.0)	5.3 (4.7–5.8)	.7
HDL cholesterol (mmol/l)†	1.3 (1.1–1.6)	1.3 (1.1–1.6)	.6
Triglycerides (mmol/l)†	1.2 (1–1.8)	1.3 (0.9–1.9)	.9
Testosterone (nmol/l)†	14.3 (11.3–17.3)	15.1 (12.2–18.8)	.08
Impaired glucose tolerance	105 (70%)	69 (66%)	.5
FINDRISC diabetes risk score†	5.3 (3.6–8.3)	3.6 (1.9–5.9)	<.001
Beta-blocker medication	25 (17%)	19 (18%)	.7
Smoking	37 (25%)	22 (21%)	.5
Schooling after grade school	31 (22%)	40 (41%)	.002
Living with a partner	121 (85%)	86 (89%)	.4
Beck's depression inventory	5 (2–8)	2 (0–5)	<.001

ED = Erectile dysfunction; FINDRISC = Finnish Diabetes Risk Score; HDL, high-density lipoprotein.

∗ Mean and SD reported for variables following normal distribution.

† Median and interquartile range reported for variables with skewed distribution.

**Table 2:** Risk factors for erectile dysfunction in univariate analysis. Cross-sectional analysis of 254 Finnish men with cardiovascular risk factors

Uric Acid	Or (95% CI)	*P*
Age	1.12 (1.07–1.17)	<.001
Body mass index	0.98 (0.92–1.04)	.5
Categorized by median (28.1 kg/m^2^)	0.97 (0.59–1.61)	.9
Systolic blood pressure	1.01 (0.99–1.02)	.3
Categorized by median (148 mmHg)	1.27 (0.77–2.10)	.4
Estimated glomerular filtration rate	0.98 (0.96–1.00)	.056
Categorized by median (90 mL/min)	0.78 (0.47–1.30)	.3
Serum uric acid	1.01 (1.00–1.01)	.4
Categorized by median (375 μmol/l)	1.22 (0.74–2.01)	.4
Total cholesterol	1.06 (0.81–1.38)	.7
HDL cholesterol	1.15 (0.62–2.14)	.7
Triglycerides	0.92 (0.67–1.28)	.6
Testosterone	0.95 (0.90–1.00)	.071
Categorized by median (14.7 nmol/l)	0.77 (0.47–1.27)	.3
Impaired glucose tolerance of any level	1.18 (0.69–2.02)	.5
Categorized in 4 groups	1.26 (0.91–1.73)	.16
FINDRISC diabetes risk score	1.15 (1.06–1.25)	<.001
Categorized by median (4 points)	1.80 (1.06–3.07)	.03
Beta-blocker medication	0.92 (0.46–1.73)	.7
Smoking	1.21 (0.66–2.20)	.5
Schooling after grade school, any level	0.41 (0.23–0.71)	<.001
Categorized in 4 groups	0.56 (0.38–0.83)	<.001
Living with a partner	0.71 (0.32–1.53)	.4
Beck's depression inventory	1.15 (1.07–1.24)	<.001
Categorized by median (3 points)	3.06 (1.71–5.49)	<.001

HDL = high-density lipoprotein.

**Table 3:** Risk factors for erectile dysfunction in multivariable analysis. Cross-sectional analysis of 254 Finnish men with cardiovascular risk factors

ED Risk Factor	Or (95% CI)	*P*
Age	1.11 (1.05–1.17)	<.001
Body mass index	1.01 (0.92–1.10)	.9
Categorized by median (28.1 kg/m^2^)	1.29 (0.67–2.45)	.4
Systolic blood pressure	1.00 (0.98–1.02)	.9
Categorized by median (148 mmHg)	1.24 (0.67–2.28)	.5
Estimated glomerular filtration rate	1.00 (0.97–1.03)	.8
Categorized by median (90 mL/min)	1.28 (0.64–2.56)	.5
Serum uric acid	1.00 (1.00–1.01)	.7
Categorized by median (375 μmol/l)	1.14 (0.59–2.19)	.7
HDL cholesterol	0.92 (0.41–2.07)	.8
Triglycerides	1.09 (0.71–1.66)	.7
Testosterone	0.95 (0.88–1.01)	.1
Categorized by median (14.7 nmol/l)	0.67 (0.35–1.30)	.2
Impaired glucose tolerance of any level	1.07 (0.55–2.11)	.8
Categorized in 4 groups	1.13 (0.75–1.70)	.6
Beta-blocker medication	0.59 (0.25–1.38)	.2
Schooling after grade school, any level	0.52 (0.33–0.83)	.005
Categorized in 4 groups	0.47 (0.24–0.91)	.025
Living with a partner	0.71 (0.32–1.53)	.4
Beck's depression inventory	1.17 (1.08–1.27)	<.001
Categorized by median (3 points)	3.46 (1.83–6.55)	<.001

HDL = high-density lipoprotein.

Differences in BMI, smoking habits, or serum testosterone levels were not statistically significant. Blood pressure levels, eGFRs, and cholesterol levels were comparable between the groups. Men with ED scored significantly higher in Beck's depression inventory (*P* < .001). No other significant differences were found between the groups.

### Uric Acid as ED Risk Factor

The results of univariate analysis are presented in Table 2. A statistically significant association (*P* < .05) for occurrence of ED was found for eGFR (both continuous and stratified by median), age, impaired glucose tolerance, serum testosterone, depressive symptoms, and level of education. These variables were included in multivariable-adjusted regression analysis to identify independent ED risk factors. CVD is a known risk factor for ED, so variables closely associated with CVD (BMI, HDL-C, triglycerides, systolic blood pressure) were also included in the model. Living conditions were not statistically significantly associated with ED in univariate analysis and were thus excluded. In multivariable analysis, Finnish Diabetes Risk Score was not an independent risk factor after inclusion of the cardiovascular risk factors already included in the score and was excluded from the multivariable analysis.

Uric acid was not associated with ED risk in univariate or multivariable analysis (multivariable-adjusted OR 1.14, 95% CI 0.59–2.19, *P* = .7) for SUA level higher than median compared to median or lesser (OR 1.00, 95% CI 0.997–1.006, *P* = .7 for trend by SUA as continuous variable). In univariate logistic regression analysis, statistically significant or borderline significant associations with ED were found for age, impaired glucose tolerance, eGFR, serum testosterone level, education level, and depressive symptoms.

The results of multivariate analysis are presented in Table 3. Education level remained an independent predictor of ED in multivariable adjusted analysis; the risk was lower in men with higher education level compared than in those with lower education level (OR 0.49, 95% CI 0.50– 0.80). Depressive symptoms as per Beck Depression Inventory remained a strong predictor for ED also in multivariable adjusted analysis (OR 1.17, 95% CI 1.08–1.27) as well as age (OR 1.11, 95% CI 1.05–1.17). Testosterone was a borderline significant predictive factor.

### Subgroup Analysis

Participant age, eGFR, glucose tolerance, testosterone, BMI, depression inventory results or presence of MetS did not modify the association between SUA and ED (Table 4). Of other ED risk factors, eGFR modified the association for testosterone; in the low eGFR group, higher testosterone level was associated with lower risk of ED, while in normal eGFR group, no risk difference by testosterone was observed (*P* for difference 0.04).

**Table 4:** Serum UA as a risk factor for ED in different subpopulations. Cross-sectional analysis of 254 Finnish men with cardiovascular risk factors

	Age greater than median (45–57 y)	Age greater than median (58–70 y)	Non-impaired glucose tolerance (n = 80)	Impaired glucose tolerance (n = 174)	Testosterone lesser than median (6.1–14.6)	Testosterone greater than median (14.7–32.9)	eGFR lower than median (50–90)	eGFR greater than median (91–115)	BMI lower than median (20.1–28.0)	BMI greater than median (28.1–53.9)	BDI lesser than or equal to median (0–3)	BDI above median (4-6)	No MetS as per to IDF criteria	MetS diagnosed as per IDF criteria
	OR (95% CI)		OR (95% CI)		OR (95% CI)		OR (95% CI)		OR (95% CI)		OR (95% CI)		OR (95% CI)	
Urate (continuous variable)	1.01 (1.00–1.01)	1.00 (0.99–1.00)	1.00 (0.99–1.01)	0.00 (0.00–0.00)	1.00 (0.99–1.01)	1.00 (1.00–1.01)	1.00 (0.99–1.01)	1.00 (1.00–1.01)	1.00 (0.99–1.01)	1.00 (0.99-1.01)	1.01 (1.00–1.01)	1.00 (0.99–1.01)	1.00 (0.99–1.01)	1.00 (1.00–1.01)
Urate (stratified by median)	1.49 (0.67–3.31)	0.67 (0.25–1.80)	0.73 (0.25–2.13)	1.00 (1.00–1.01)	0.76 (0.30–1.98)	1.58 (0.68–3.70)	1.10 (0.44–2.74)	1.48 (0.62–3.51)	1.11 (0.45–2.75)	0.95 (0.41–2.22)	0.33 (0.13–0.80)	1.68 (0.58–4.85)	0.56 (0.17–1.84)	1.66 (0.69–4.03)
*P*∗	.11		.16		.3		.3		.8		.5		.097	

Any level of erectile dysfunction, OR (95% CI), multivariable adjusted.

BMI = body mass index; eGFR = estimated glomerular filtration rate; MetS = metabolic syndrome; UA = uric acid.

∗ *P* value for interaction term between SUA and the variable of interest.

Age, BMI, or impaired glucose tolerance did not modify the risk association for uric acid. Age did modify the risk association for testosterone. Inverse association between testosterone and ED was only found in men aged 59 years or older (*P* for interaction = 0.032). BMI did not modify any ED risk factor.

An association between elevated triglycerides and increased risk of ED was found in a subpopulation with impaired glucose tolerance suggesting metabolic disturbance. Notably, none of the performed subgroup analysis modified the findings for SUA. No association to risk of ED was found in any subgroup.

### Sensitivity Analysis

Sensitivity analysis was performed by adjusting the analysis for total propensity score for ED. In this analysis, no clear risk association was observed between SUA and ED; OR 1.13 (95% CI 0.66–1.94). This supported the results of the multivariable analysis. Considering all the aforementioned risk factors, SUA level is not an independent risk factor of ED.

## Discussion

Our study does not support hyperuricemia as an ED risk factor. Hyperuricemia and MetS are closely connected, and MetS in itself is also associated with ED and CVD.^7^ Hyperuricemia can be managed through pharmaceutical and dietary interventions, thus if proven to be an ED risk factor would provide a new avenue to decrease risk for the condition. However, such an association was not found when confounding factors such as GFR were considered.

ED is a neurovascular disorder regulated by hormones. Neurologic conditions often associated with ED include multiple sclerosis, Parkinson's disease, Alzheimer's disease, and spinal cord injuries of different origins.^27^^,^^28^ Vascular changes, often preceded by and presenting as endothelial dysfunction, are the most significant factor in developing ED.^8^ Hormonal disorders resulting in hypogonadism and subsequent ED are often associated with MetS.

ED has often been discovered to be the first symptom of CVD. These 2 conditions share a similar pathogenesis, and ED is typically present before other clinical cardiovascular events.^29^^,^^30^ Considering the public health impact of CVD, it is sensible to look closer into populations with ED: Novel physical or biochemical markers associated with ED could be useful for assessing the risk not only for CVD but potentially also for stroke and all-cause mortality.

The established signs of MetS include central obesity, hypertension, hyperlipidemia (specifically elevated serum triglycerides), and impaired glucose tolerance. These all have also been associated with ED.^2^^,^^31^^,^^32^ Typically in MetS, the renin-angiotensin system is also activated leading to activation of xanthine oxidase pathway which is central for purine metabolism.^10^ Excessive production of uric acid and ensuing highly reactive free radicals lead to increased oxidative stress and systemic inflammation.

An association between hyperuricemia and ED would be clinically significant as it would have major implications for better understanding the prevention and consequences of the obesity epidemic.

Large amounts of xanthine oxidase, among other oxidases and oxygenases, are found also in vascular endothelium. Their activation through angiotensin II impairs the nitric oxide signaling and thereby inhibits vasodilatation.^12^ The same phenomenon is seen in arteriosclerosis and cardiac insufficiency and is also largely responsible for the lack of peripheral vasodilatation after smoking.^33^^,^^34^ This effect can be partially reversed by allopurinol, which decreases the SUA concentration.^35^ Dysfunctional vasodilatation in small vessels can manifest as ED and provides a possible link between ED and SUA.

Research in the field of purine metabolism is scarce compared with that of glucose and lipid metabolism. The use of medical interventions regarding purine metabolism is less frequent as well. Regardless, evidence on an association between purine metabolism and different manifestations of both MetS and CVD exists. However, the exact underlying mechanisms and causality are still not completely understood and conflicting studies have been published. ED is an early clinical manifestation of both CVD and MetS. It is therefore tempting to seek targets for pharmacologic intervention that could modify the pathogenesis of these conditions. Purine metabolism that is SUA levels is one such potential target that has been also previously addressed in the literature.

In contrast to our study, Salem et al^36^ reported an association between SUA and ED. They also proposed SUA as an independent risk factor for ED. MetS (and thus disturbances of purine metabolism) and CVD share several risk factors: these include aging, obesity, smoking, impaired glucose tolerance, and hypertension. However, when assessing SUA level as a risk factor, it is necessary to take kidney function into consideration because the elimination of SUA depends on it. In the study by Salem et al,^36^ GFR was not accounted for and the mean SUA levels of the study population differed significantly from normal values, as Reis et al^18^ have pointed out. These remarks may well explain the fundamentally different results from ours.

ED has been established as a possible first sign of CVD. Therefore, the possibility of recognizing the risk of ED earlier based on SUA would greatly benefit the prevention of CVD. The association of SUA and ED has been analyzed in related subgroups. Solak et al^37^ studied the association of SUA and ED in a population of 312 patients with CVD. It is worth noting that the ED and non-ED populations were quite different regarding age, GFR, and prevalence of diabetes. After adjusting the analysis for relevant risk factors, including those mentioned, no association could be found.

Aribas et al studied the association of SUA and ED in hypertensive patients.^38^ After adjusting the analysis strictly, they established SUA as an independent risk factor of ED. In addition to the known risk factors they also adjusted their model for a wide array of medications and an association of SUA and ED was found despite a small study population. This result is biologically plausible considering that hyperuricemia contributes to endothelial dysfunction, which plays a role in the pathogenesis of hypertension.^39^^,^^40^ In these patients, there is an ongoing process affecting the cardiovascular system and seemingly erection as well. The study population differed from ours, as normal blood pressure was not an exclusion criterion in our study. This probably leads to differing results.

MetS is associated with a wide disruption of the endocrine system, which is partly involved in modulating all risk factors of ED and CVD and possibly directly ED as well. Gao et al^41^ analyzed a large group of Chinese men (n = 1,365) and included several sex-related hormonal markers (testosterone, follicle stimulating hormone, luteinizing hormone, SHBG, free androgen index, HbA1c, fasting glucose) in their study. Adjusted for all other markers, they found an independent association between SUA and ED. Consequently, based on this large study, we can safely assume that including a comprehensive array of endocrine markers does not modify the association. Unfortunately, some of the risk factors such as smoking and GFR were left out, so further conclusions are not possible. Although we were not able to adjust the analysis for all endocrine risk factors studied by Fengbin et al, we were able to adjust for serum testosterone, impaired glucose tolerance and further for BMI, smoking, and GFR. After such adjustment, we did not find significant association between SUA and ED, arguing against SUA being an independent risk factor for ED.

It has to be noted that none of the studies included depression in their statistical analysis. Solak et al excluded patients with depression, which alleviates this problem. Based on our results, depression is the most significant predictor of ED, and therefore, taking depressive symptoms into account is imperative in future studies on risk of ED.

Our study was population based, and the sample can be considered representative of the background population. The study population consists entirely of Caucasian men, so generalizability regarding men of other ethnicities is uncertain. We were able to include a broad array of cardiovascular risk factors and comorbidities to our analysis. Sexually inactive men were excluded from the study population, which could skew the results as inactivity may be caused by ED. Our information on ED was collected with a validated instrument, the IIEF-5 questionnaire.^23^

Unfortunately, only a minority of the population could be included to guarantee satisfactory data on both SUA and ED, reducing statistical power to detect a difference in our analysis. The excluded population had similar characteristics to the included population, thus exclusions were not likely to cause systematic bias owing to any measured factor. The subgroup analyses can be considered explanatory in nature owing to the small population.

The difference of SUA between men with and without ED was small in our study population compared with some of the earlier studies on the subject. Some of these studies found a clear and statistically significant difference for SUA between the groups with and without ED.^36^, ^37^, ^38^ In our highly comparable groups and in the report by Gao et al,^41^ such a clear difference could not be found.

Nevertheless, our quantitatively limited study population degrades the sensitivity of analysis, and therefore, a larger study could have yielded additional statistically significant findings: a post hoc power calculation indicated that a study as large as 3,000 subjects would have been needed to yield a statistically significant difference in SUA values, which undermines the clinical utility of using SUA as a biomarker for risk of ED.

## Conclusion

Based on this quantitatively limited but well-characterized population, we can conclude that SUA is not independently associated with onset of ED. Taking eGFR into consideration does not modify the result.

## Statement of authorship

Antti T. Tuokko: Formal Analysis, Data Curation, Writing - Original Draft; Teemu J. Murtola: Formal Analysis, Writing - Original Draft, Supervision; Päivi E. Korhonen: Methodology, Investigation, Resources, Writing - Review & Editing; Antti J. Kaipia: Conceptualization, Writing - Review & Editing, Supervision
